# Supplementary material for: c-Cbl Regulates Murine Subventricular Zone-Derived Neural Progenitor Cells in Dependence of the Epidermal Growth Factor Receptor
Source: Cells. 2023 Oct 3;12(19):2400. doi: 10.3390/cells12192400 (PMC10572332; doi:10.3390/cells12192400)
Supplement: Supplementary file 1 [file cells-12-02400-s001.zip › cells-2564297-supplementary.pdf]

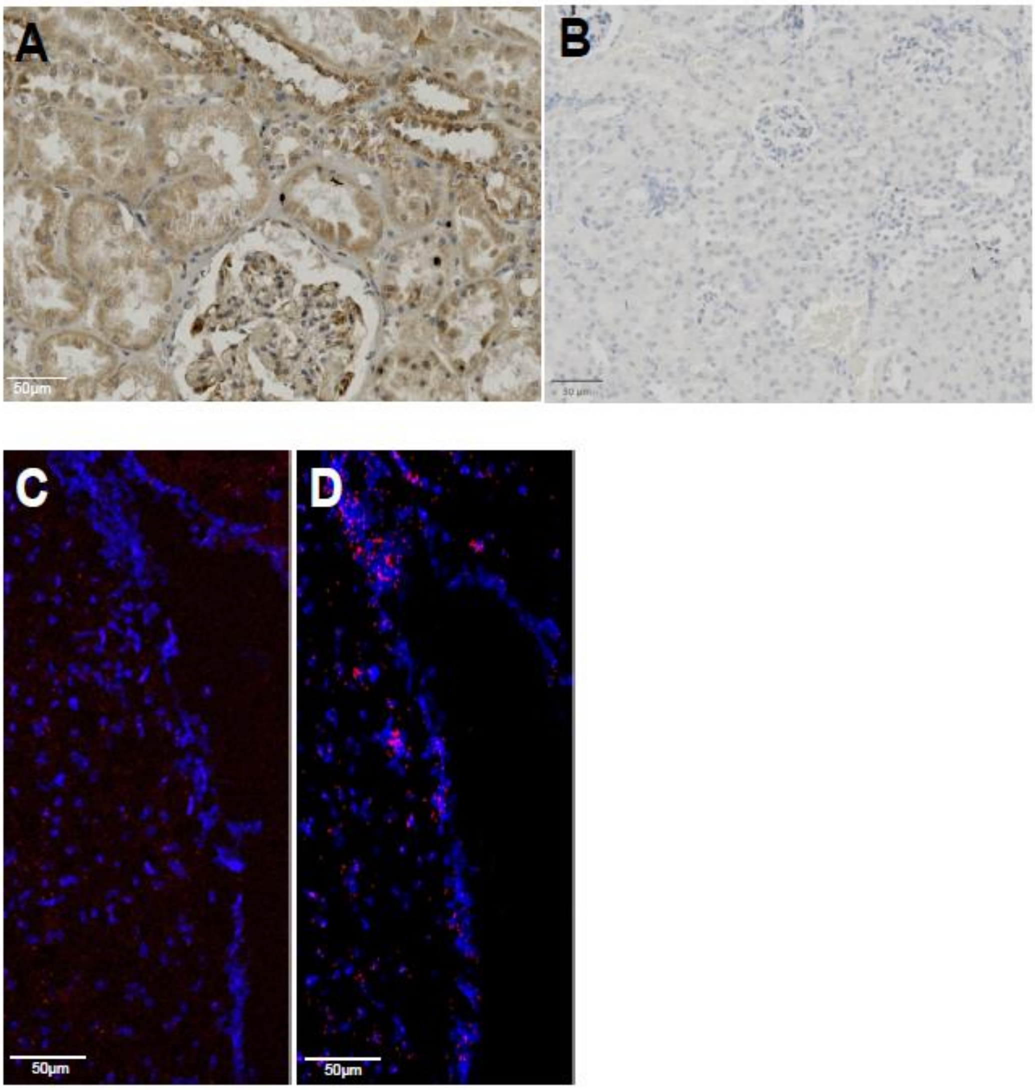

Figure S1. c-Cbl expression.

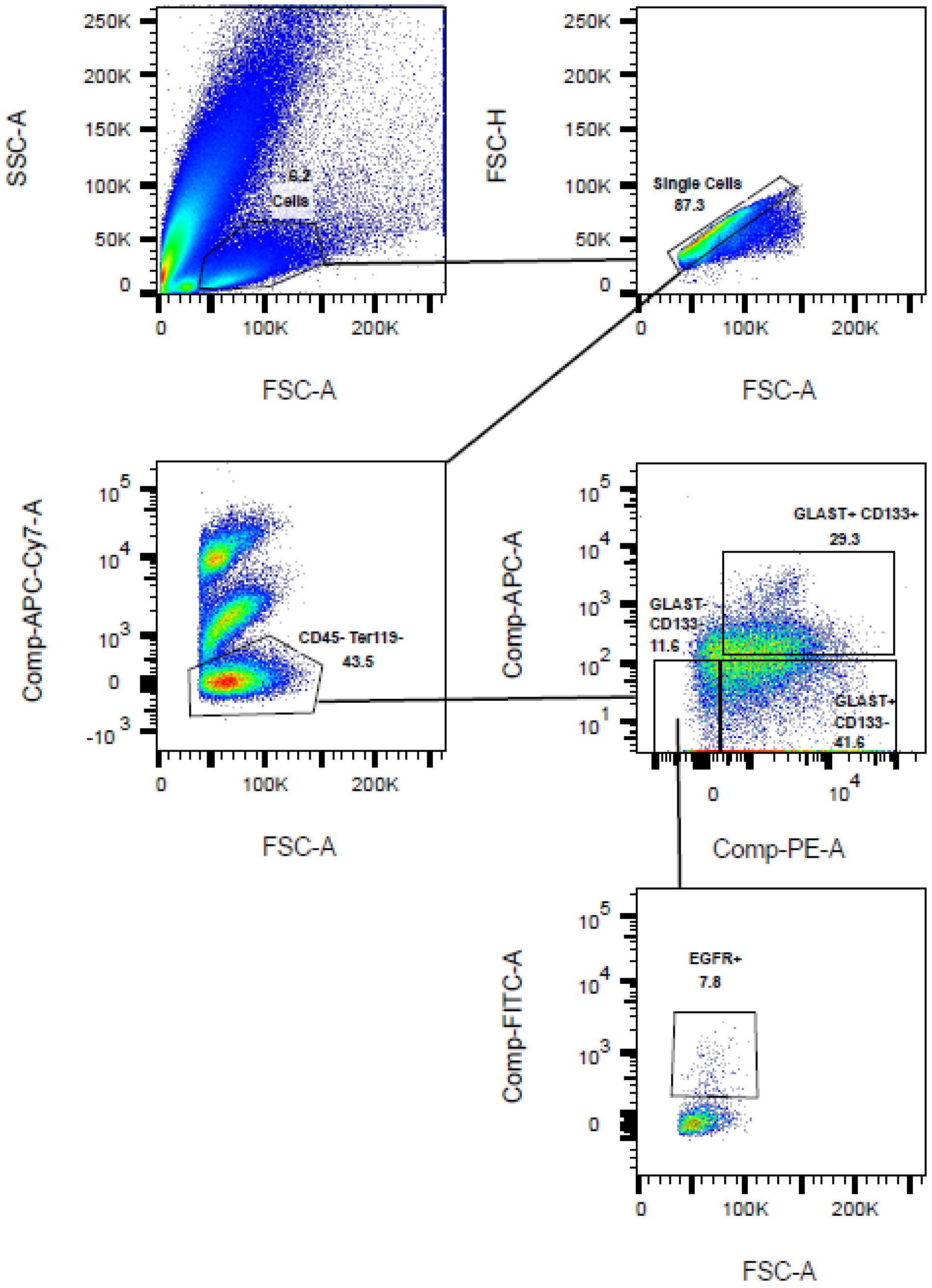

Figure S2. FACS procedure for the isolation of NSCs, TAPs, and astrocytes from the SVZ in mice.
